# OXER1 and RACK1-associated pathway: a promising drug target for breast cancer progression

**DOI:** 10.1038/s41389-020-00291-x

**Published:** 2020-12-11

**Authors:** Mirco Masi, Enrico Garattini, Marco Bolis, Daniele Di Marino, Luisa Maraccani, Elena Morelli, Ambra A. Grolla, Francesca Fagiani, Emanuela Corsini, Cristina Travelli, Stefano Govoni, Marco Racchi, Erica Buoso

**Affiliations:** 1 Dipartimento di Scienze del Farmaco, Università Degli Studi di Pavia, Viale Taramelli 12/14, 27100 Pavia, Italy; 2 Scuola Universitaria Superiore IUSS, Piazza della Vittoria 15, 27100 Pavia, Italy; 3 Laboratory of Molecular Biology, Istituto di Ricerche Farmacologiche Mario Negri IRCCS, via Mario Negri 2, 20156 Milano, Italy; 4 Functional Cancer Genomics Laboratory, Institute of Oncology Research, USI, University of Southern Switzerland, 6500 Bellinzona, Switzerland; 5 Bioinformatics Core Unit Institute of Oncology Research, Swiss Institute of Bioinformatics, 1000 Lausanne, Switzerland; 6 Department of Life and Environmental Sciences, New York-Marche Structural Biology Center (NY-MaSBiC), Polytechnic University of Marche, Ancona, Italy; 7 Department of Pharmaceutical Sciences, University of Piemonte Orientale, via Bovio 6, 28100 Novara, Italy; 8 Laboratory of Toxicology, Dipartimento di Scienze Politiche ed Ambientali, Università Degli Studi di Milano, Via Balzaretti 9, 20133 Milano, Italy

**Keywords:** Phosphoinositol signalling, Breast cancer, Hormone receptors

## Abstract

Recent data indicate that receptor for activated C kinase 1 (RACK1) is a putative prognostic marker and drug target in breast cancer (BC). High RACK1 expression is negatively associated with overall survival, as it seems to promote BC progression. In tumors, RACK1 expression is controlled by a complex balance between glucocorticoids and androgens. Given the fact that androgens and androgenic derivatives can inhibit BC cell proliferation and migration, the role of androgen signaling in regulating RACK1 transcription in mammary tumors is of pivotal interest. Here, we provide evidence that nandrolone (19-nortosterone) inhibits BC cell proliferation and migration by antagonizing the PI3K/Akt/NF-κB signaling pathway, which eventually results in RACK1 downregulation. We also show that nandrolone impairs the PI3K/Akt/NF-κB signaling pathway and decreases RACK1 expression via binding to the membrane-bound receptor, oxoeicosanoid receptor 1 (OXER1). High levels of OXER1 are observed in several BC cell lines and correlate with RACK1 expression and poor prognosis. Our data provide evidence on the role played by the OXER1-dependent intracellular pathway in BC progression and shed light on the mechanisms underlying membrane-dependent androgen effects on RACK1 regulation. Besides the mechanistic relevance, the results of the study are of interest from a translational prospective. In fact, they identify a new and actionable pathway to be used for the design of innovative and rational therapeutic strategies in the context of the personalized treatment of BC. In addition, they draw attention on nandrolone-based compounds that lack hormonal activity as potential anti-tumor agents.

## Introduction

Breast cancer (BC) is a heterogeneous disease due to variable histological subtypes and differences in response to therapy and clinical outcome^1^. In human BC cells, the receptor for activated C kinase 1 (RACK1) has been identified as a possible prognostic marker and drug target^2^ due to its critical role in cancer cell migration and invasion^3–6^. Changes in RACK1 levels have been found to subvert physiological functions, leading to the development and maintenance of several BC hallmarks^6–9^. Indeed, data mining analyses (Chin, 130; Bertucci 266; Booser, 508) revealed that elevated RACK1 expression negatively correlates with overall survival (OS)^10^, thus indicating that RACK1 over-expression associates with poor clinical outcome^2^.

A complex hormonal balance between glucocorticoids and androgens has been demonstrated to control RACK1 expression in both the immune^11–16^ and the cancer context^5,9^. Consequently, based on the hormone-related nature of many BC types, a deeper understanding of RACK1 transcriptional regulation is of pivotal interest^17^. Accumulating evidence suggests a role for androgen signaling in BC^18^. In this regard, literature data report that the adrenal hormone dehydroepiandrosterone (DHEA) and its endogenous androgenic derivative 7β-Hydroxy-epiandrosterone (7β-OH-EpiA) exert a protective role against cancer by inhibiting cell proliferation and migration^19–21^. Notably, different testosterone derivatives have been demonstrated to inhibit cell proliferation, thus suggesting that the development of testosterone-based anticancer drugs, lacking for hormonal activity, may represent a major challenge in steroid medicinal chemistry^22,23^. Increasing evidence indicates that androstanes and their structural analogs display a versatile anti-proliferative action against a broad variety of cancer cell lines, including prostate, breast, cervix, ovarian, leukemia, melanoma, colon, and gastric cancers^23–29^. As DHEA regulates RACK1 expression through an androgenic mechanism in the immune context^13–16^, we investigated whether the synthetic anabolic-androgenic steroid, nandrolone (19-nortestosterone) was able to modulate RACK1 expression in BC cells. Here we report that RACK1 is significantly downregulated by synthetic steroids that bind oxoeicosanoid receptor 1 (OXER1), a member of the G-protein-coupled receptors (GPCR) family involved in the biological action of the arachidonic acid metabolite 5-oxo-eicosatetraenoic acid (5-oxoETE)^30^. OXER1 is involved in both inflammation and oncogenesis, but its role and its significance in BC progression are just emerging^31^. Indeed, immunohistochemical analyses revealed that membrane staining for OXER1 is higher in tumor compared to non-cancerous tissues^32^. In accordance, BC TCGA data analysis revealed that high OXER1 expression correlated with estrogen- (ER) and progesterone receptors (PR) status, suggesting that OXER1 is a new and attractive target for pharmacological intervention. Here, we demonstrate that OXER1 silencing significantly inhibits BC cell proliferation and migration by disrupting PI3K/Akt/NF-κB pathway, thereby leading to RACK1 downregulation. Therefore, RACK1 transcriptional regulation and OXER1 function in BC cell proliferation and migration highlight the existence of a novel molecular mechanism that could provide new and relevant drug targets for BC treatment.

## Materials and methods

### Chemicals, culture media, and supplements

Wortmannin (Pubchem CID: 312145), Testosterone (Pubchem CID: 6013), Testosterone-BSA-FITC (T5771), Nandrolone (Pubchem CID: 9904), DHEA (Pubchem CID:5881), Flutamide (Pubchem CID:3397), BAY 11-7082 (Pubchem CID: 5353431), G418 (Pubchem CID: 123865), cell culture media and all supplements were obtained from Sigma Aldrich (St. Louis, MO, USA). Anti-RACK1 (sc-17754), anti-c-Rel (sc-6955), anti-IκBα (sc-371), and anti-phospho (Ser32/36) IκBα (sc-101713) were obtained from Santa Cruz Biotechnology (Dallas, TX, USA). Anti-β-tubulin (T0198) was obtained from Sigma-Aldrich. Anti-Akt (#9272) and anti-phospho (Ser473) Akt (#9721) were purchased from Cell Signaling Technology (Danvers, MA, USA). Anti-AR 441 (ab9474) was obtained from Abcam (Cambridge, UK). Anti-β-actin (612656) and anti-Lamin A/C (612162) were obtained from BD Biosciences (Franklin Lakes, NJ, USA). Anti-OXER1 (orb227698) was obtained from Biorbyt (Cambridge, UK). Host-specific peroxidase-conjugated IgG secondary antibodies were purchased from Thermo Scientific Inc. (Waltham, MA, USA).

### Cell cultures and treatments

MCF7 and MDA-MB-231 (ATCC^®^, Rockville, MD) are maintained as described in refs. ^3,5^. To perform testosterone, nandrolone, and testosterone-BSA-FITC treatments, MCF7 and MDA-MB-231 cells were cultured in complete medium without phenol red and supplemented with 5% dextran-coated charcoal-treated fetal bovine serum (DCC-FBS). MCF7 stable clone overexpressing wild-type RACK1 (termed RWT) was transfected with pcDNA3.1myc/His/Neo plasmid carrying RACK1 cDNA kindly provided by Professor Ceci Marcello^33^. Clone selection was maintained with 500 μg/mL G418. Treatment details are given in figure legends.

Compounds used for treatments were dissolved in DMSO (Sigma Aldrich Italia, Cas N° 67-68-5, purity 99.9%) at concentration of 50 mM and frozen at −20 °C in stock aliquots. Stocks were diluted at final concentrations in culture media at the time of use (final concentration of DMSO in the culture medium <0.1%). Control cells were treated with the same amount of DMSO.

### Luciferase assays

Plasmids were purified with the HiSpeed^®^ Plasmid Midi Kit (Qiagen, Valencia, CA) and DNA was quantified by Quantus™ Fluorometer (Promega, Madison, WI). Δ1, Δ2, Δ7, and Δ11 luciferase plasmids described in ref. ^34^ were transfected with Lipofectamine^®^ 2000 (Thermo Fisher, Waltham, MA, USA) as detailed in ref. ^5^. After treatment, cells were lysed and analyzed following Dual-Luciferase Reporter Assay System specifications (Promega, Madison, WI). Luminescence was measured with a 20/20n Luminometer with 10 s integration (Turner BioSystems, Sunnyvale, CA).

### qPCR

2 × 10^6^ cells were seeded in 60 mm dishes. Total RNA was extracted using RNeasy Plus Mini Kit (Qiagen, Valencia, CA, USA) and RNA quantification was performed with Quantus™ Fluorometer (Promega, Madison, WI). Qiagen QuantiTect reverse transcription kit was used for cDNA synthesis. qPCR was performed with QuantiTect Sybr Green PCR kit and RACK1 and RpL6 primers, all provided by Qiagen as indicate in refs. ^5,14–16^. RpL6 was used as endogenous reference control^5,34^ and transcripts quantification was performed with 2^(−ΔΔCT)^ method^5,16^.

### Subcellular fractionation

In brief, 3.5 × 10^6^ MCF7 were seeded in 100 mm dishes and treated for 24 h with 100 nM Nandrolone or Testosterone-BSA-FITC and cellular fractionation was conducted as described in detail in ref. ^35^.

### Western blot analysis

The expression of OXER1, AR, Akt, p-Akt, IkB-α, p-IkB-α, Lamin A/C, c-Rel, RACK1, β-actin, and β-tubulin in cell homogenates was assessed by Western blot analysis as described in detail in ref. ^5^. After Western blot acquisition, bands optical analysis was performed with the ImageJ program (W. Rasband, Research Service Branch, National Institute of Mental Health, National Institutes of Health, Bethesda, MD and Laboratory for Optical and Computational Instrumentation, University of Wisconsin). Bands relative densities were expressed as arbitrary units and normalized over control sample run under the same conditions.

### Immunofluorescence

5000 MDA-MB-231 or 10.000 MCF7 cells per well were seeded on sterile slides coated with poly-L-Lysine in 24 multi-well plates. Cells were fixed and stained following protocol from ref. ^36^. Acquisition was performed with confocal laser scanning SP5 by “Centro Grandi Strumenti” - Pavia.

### Molecular modeling and docking

The three-dimensional (3D) structure of OXER1 was obtained using the he GPCR-Sequence-Structure-Feature-Extractor (SSFE) web server (10.1093/nar/gkx399). The GPCR-SSFE 2.0 allows a multi-template approach for the modeling of both transmembrane (TM) helices and extracellular and intracellular loops. The templates that were selected for the modeling of the TM helices of OXER1 are reported in supplementary Table 1.

Before running the molecular docking the OXER1 structural model was regularized with 5000 steps of steepest descent energy minimization using the AMBER 14 software (University of California, San Francisco, 2014).

The structures of nandrolone and testosterone have been prepared for the docking using LigPrep (LigPrep, Schrödinger, LLC, New York, NY, 2015), thus generating all the possible tautomers and protonation states in the pH range 6.0–8.0. The molecular docking search area was placed at the centre of the transmembrane helices facing the extracellular side. The grid has been generated using the grid generation tool of Glide (Glide, Schrödinger, LLC, New York, NY, 2015) with default settings. The Glide SP scoring function was used to run docking calculations and to score the predicted docking poses (Glide, Schrödinger, LLC, New York, NY, 2015).

### Small Interference RNA and shRNA

Commercially available MISSION^®^ esiRNAs for AR (EHU025951) and OXER1 (EHU142961) were obtained from Sigma Aldrich and transfected with Lipofectamine^®^ RNAiMAX for knock-down experiments. In brief, 2 × 10^5^ MDA-MB-231 cells or 3 × 10^5^ MCF7 cells were seeded in 6 multi-well plates and, 48 h after transfection, were treated as described in figure legends.

The stable RACK1-silenced cell line (shRACK1) was obtained by lentiviral infection. The lentiviral particles were produced as described elsewhere^37^ in Hek293T cells by a second-generation packaging plasmid system containing a GIPZ_GFP human RACK1 Lentiviral shRNA plasmid or a scramble vector (RHS4531-EG10399; GE-Dharmacon). In brief, Hek293T cells were transfected by Lipofectamine^®^ with GIPZ (scramble or shRACK), pPAX and pMDG vectors. After 48 h, cell medium was collected, filtered and centrifuged for 2 h at 70,000 × *g*. Viral particles were re-suspended and used to infect MCF7 cells, after virus titration. Infected cells were flow sorted for high positive GFP fluorescence (S3e Cell Sorter BIO-RAD). Silencing was monitored by Western blot analysis.

### Scratch wound healing assay

Cells were seeded in a 6-well plate, grown to confluence and incubated in 5% DCC-FBS medium containing nandrolone (100 nM) or testosterone-BSA-FITC (100 nM). Scratch wound healing assays and image analysis were performed as described in refs. ^5,19^.

### Viability and cell proliferation

Cells were seeded in 96-multiwell culture plates at a concentration of 5000 cells/well in a final volume of 100 μL. Viability and proliferation assay was assessed by Cell Proliferation Kit I (MTT) (Sigma Aldrich) a colorimetric assay based on the reduction of MTT [3-(4,5-dimethylthiazol-2yl)-2,5-diphenyl-tetrazolium bromide] and readings were made through Synergy HT microplate reader (Bio Tek Instruments, Milan, IT).

Cells were seeded and after 24 h (*t* = 0) they were treated according to concentrations and timing reported in detail in figure legends.

### Colony assays

Cells were seeded in 6 multi-well plates at a concentration of 2000 cells/well and colony assay was performed as described in ref. ^38^.

### Cytofluorimetry

MCF7 cells were seeded at a concentration of 10^6^ cells/dish grown in complete DMEM and subsequently were starved for 24 h in DMEM without FBS. After 24 h of starvation, MCF7 cells were grown for 24 h in complete DMEM medium and then treated with 100 nM nandrolone for 24 h. Pellets were washed in ice-cold PBS, resuspended in ice-cold ethanol 70% and stored at −20 °C overnight. Pellets were resuspended in ice-cold PBS with the RNase (100 µg/mL) addition and incubated at 37 °C for 1 h. EDTA 5 mM and propidium iodide (100 µg/mL) were added to the suspension and the solution was incubated on ice and protected from light for 30 min before FACS acquisition (protocol adapted from ref. ^19^). FACS analysis was performed with CyFlow Space^®^ cytofluorometer (Sysmex Partec Italia S.R.L., Cornaredo, IT) and data were analyzed with the FlowMax^®^ software (Partec Inter AG, Freienbach, CH).

### Statistical analysis

Statistical analyses were conducted with GraphPad Prism version 7 (GraphPad Software, San Diego, CA, USA). Statistical differences were determined by analysis of variance (ANOVA) followed, when significant, by an appropriate post hoc test, as indicated in the figure legends. In all reported statistical analyses, effects were designated as significant if the *p* value was < 0.05.

## Results

### Androgens negatively regulate RACK1 expression by an independent AR mechanism

Since BC is mostly a hormone-related tumor^18^ and RACK1 expression regulation is under control of a complex hormonal balance^12^, we investigated RACK1 involvement in BC progression and its expression following androgen treatment. To this purpose, we used the MCF7 cell line, a well-established model to study hormone response and resistance pathways, given its androgen receptor (AR), PR, and glucocorticoid receptor (GR) positive profile^39^. In accordance with the tight correlation demonstrated between RACK1 expression as well as cell proliferation and migration in BC^3–5,40^, cell growth is significantly increased in MCF7 cells stably over-expressing RACK1 (RWT) and completely abolished in RACK1-silenced cells (shRACK1), compared to controls (Suppl. Fig. S1A, B). In addition, RACK1 depletion significantly affects MCF7 migration (Suppl. Fig. S1C). Consistent with the literature data reporting DHEA-mediated regulation of RACK1 expression and inhibition of cell proliferation/migration^19,20^, MCF7 cells treated with increasing concentrations of DHEA showed a significant reduction in both cell proliferation (Suppl. Fig. S2A–B) and RACK1 expression (Suppl. Fig. S2C, D).

Considering that DHEA effects on RACK1 regulation and cell proliferation/migration are mediated by its endogenous androgenic derivatives^21^, MCF7 cells were treated with nandrolone, which is able to regulate RACK1 expression in different cellular contexts^9,12,15^ and is not subjected to aromatase-mediated intracrinological androgen-to-estrogen conversion, unlike other androgenic derivatives^41^.

Nandrolone-mediated RACK1 transcriptional regulation was investigated in terms of RACK1 promoter activity, mRNA and protein levels, showing that 100 nM nandrolone strongly reduced RACK1 expression (Suppl. Fig. S2E–G).

**Fig. 1:**
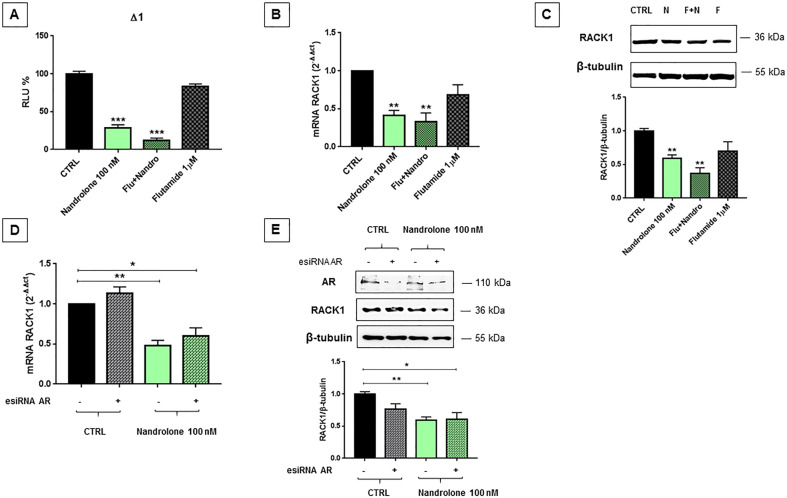
Nandrolone effect on RACK1 expression. **A**–**E** Nandrolone effect on RACK1 expression is AR-independent. **A** MCF7 cells transfected with Δ1 were treated for 16 h with 100 nM nandrolone, 1 μM flutamide, pretreated for 1 h with 1 μM flutamide and subsequently 100 nM nandrolone was added. Vehicle controls were treated with DMSO < 0.1% (CTRL). Luciferase activity was expressed as RLU% and compared to CTRL values assumed at 100%. Each bar represents the mean±SEM of three independent experiments, in quadruplicate. Statistical analysis was performed with Dunnett’s test, with ***p < 0.001. **B**–**C** MCF7 cells were treated for 16 h or 24 h with 100 nM nandrolone, 1 μM flutamide or pretreated for 1 h with 1 μM flutamide and subsequently 100 nM nandrolone was added. Vehicle controls were treated with DMSO < 0.1% (CTRL). **B** RACK1 mRNA expression analysis was performed by real-time PCR. Value bars represent the mean ± SEM of four independent experiments. **C** RACK1 expression was analyzed by Western Blotting. The image is a representative Western Blot. Results are shown as ratio RACK1/β-tubulin ± SEM of five independent experiments. **B**–**C** Statistical analysis was performed with Dunnett’s multiple comparison test, with **p < 0.01. **D**–**E** MCF7 cells were silenced for 48 h with AR esiRNA and subsequently treated for 16 or 24 h with 100 nM nandrolone for real-time PCR or Western Blotting respectively. Vehicle controls were treated with DMSO < 0.1% (CTRL). **D** RACK1 mRNA expression analysis was performed by real-time PCR. Value bars represent the mean ± SEM of three independent experiments. **E** The image is a representative Western Blot result. Results are shown as ratio RACK1/β-tubulin ± SEM of three independent experiments. **D**–**E** Statistical analysis was performed with Tukey’s multiple comparison test, with *p < 0.05, **p < 0.01 vs CTRL.

To evaluate AR involvement in RACK1 expression, MCF7 cells were pre-treated for 1 h with flutamide^42^, a nonsteroidal antiandrogen antagonist for intracellular AR, in order to abolish nandrolone-reduced RACK1 expression. Our data showed that flutamide was not able to counteract nandrolone-induced RACK1 down-regulation as demonstrated by Δ1 luciferase activity, mRNA and protein results (Fig. 1A–C), suggesting that nandrolone effect on RACK1 regulation is AR-independent. The AR-independent effect of nandrolone was confirmed through AR knockdown, suggesting the involvement of an extracellular membrane-bound AR (mAR) (Fig. 1D, E). Indeed, a large amount of evidence highlighted an alternative, membrane-initiated androgen mechanism that involves rapid signaling via specific kinases and induces a decrease in cell growth and migration by dynamic modulation of the cytoskeleton^32,43–47^.

### Nandrolone downregulates RACK1 expression by antagonizing the PI3K/Akt/NF-κB pathway

MCF7 cells treated with increasing concentration of wortmannin^48^ and BAY 11-7085^49^ showed that RACK1 was significantly downregulated with both treatments in a dose-concentration dependent manner. This demonstrates that RACK1 expression is regulated by the PI3K/Akt/NF-κB pathway (Suppl. Fig. S3A, B), in line with our previous observations^34^.

**Fig. 2:**
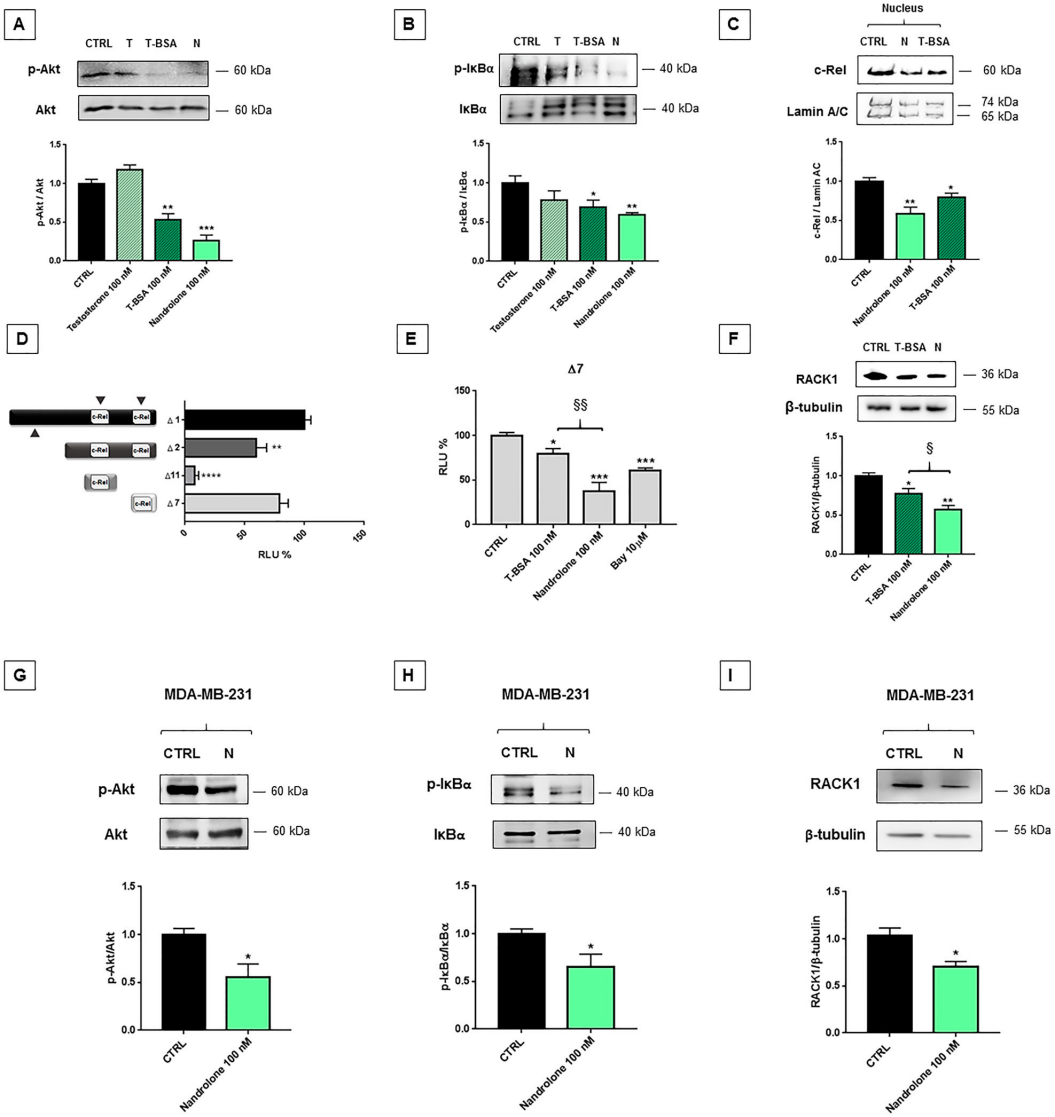
Nandrolone effect on PI3K/Akt/NF-κB pathway and RACK1-related expression. **A**–**F** Nandrolone reduces PI3K/Akt/NF-κB signaling pathway and RACK1-related expression. **A**–**B** MCF7 cells were treated for 24 h with 100 nM of testosterone (T), testosterone-BSA-FITC (T-BSA) or nandrolone (N). Vehicle controls were treated with DMSO < 0.1% (CTRL). The images are representative Western Blots. Results are shown as p-Akt/Akt (**A**), p-IκBα/IκBα (**B**) ratios ± SEM of four independent experiments. **C** Analysis of c-Rel expression in nucleus fraction of MCF7 cells treated for 24 h with 100 nM of testosterone-BSA-FITC (T-BSA) or nandrolone (N). Vehicle controls were treated with DMSO < 0.1% (CTRL). The image is a representative Western blot and results are shown as c-Rel/Lamin A/C ratio. Each value in the graph represents the mean±SEM of four independent experiments. **A**–**C** Statistical analysis was performed with Dunnett’s multiple comparison test with *p < 0.05, **p < 0.01, ***p < 0.001. **D** MCF7 cells were transiently transfected with Δ1, Δ2, Δ7, and Δ11 RACK1 promoter constructs. Δ1 is the longest construct available and contains all three c-Rel sites. Δ2 luciferase reporter construct contains only the two *cis* c-Rel sites whereas Δ11 and Δ7 contain one of the two *cis* c-Rel sites as described in ref. ^34^. The basal activity of these luciferase reporter constructs was evaluated through luciferase assay. Luciferase activity was expressed as RLU% and compared to Δ1 values assumed at 100%. Each bar represents the mean ± SEM of three independent experiments, in quadruplicate. Statistical analysis was performed with Dunnett’s test, with **p < 0.01, ****p < 0.0001. **E** MCF7 cells were transiently transfected with Δ7 and subsequently were treated for 16 h with 100 nM of testosterone-BSA-FITC (T-BSA), nandrolone (N) or with 10 µM BAY 11-7085. Vehicle controls were treated with DMSO < 0.1% (CTRL). Luciferase activity was expressed as RLU% and compared to CTRL values assumed at 100%. Each bar represents the mean±SEM of three independent experiments, in triplicate. Statistical analysis was performed with Tukey’s test, with **p < 0.01, ****p < 0.0001 vs CTRL and with ^§§^*p* < 0.01 vs T-BSA. **F** MCF7 cells were treated for 24 h with 100 nM of testosterone-BSA-FITC (T-BSA) or nandrolone (N). Vehicle controls were treated with DMSO < 0.1% (CTRL). The images are representative Western Blots. Results are shown as RACK1/β-tubulin ratio ± SEM of four independent experiments. Statistical analysis was performed with Tukey’s test, with **p < 0.01 vs CTRL and with ^§^*p* < 0.05 vs T-BSA. **G**–**I** MDA-MB-231 cells were treated for 24 h with 100 nM nandrolone. Vehicle controls were treated with DMSO < 0.1% (CTRL). The images are representative Western Blots. Results are shown as p-Akt/Akt (**G**), p-IκBα/IκBα (**H**) and RACK1/β-tubulin (**I**) ratios ± SEM of three independent experiments. Significance was set at *p* < 0.05 by the Student’s *t*-test (*p < 0.05).

To assess whether nandrolone-induced RACK1 down-regulation was related to its PI3K/Akt/NF-κB antagonism and its effect was mAR-mediated, MCF7 cells were treated with 100 nM of testosterone, testosterone-BSA-FITC or nandrolone^50^. Only nandrolone and testosterone-BSA-FITC induced a comparable and significant decrease in Akt phosphorylation (Fig. 2A), which correlated with a lower NF-κB activation as demonstrated by the reduction in IκBα phosphorylation (Fig. 2B) and c-Rel nuclear translocation (Fig. 2C). For this reason, we transiently transfected MCF7 cells with deletion mutants of a luciferase reporter construct (Δ1, Δ2, Δ7, and Δ11) involving three distinct c-Rel binding sites located inside the *RACK1* promoter^34^. Δ7 had a basal activity comparable to Δ1, indicating that this c-Rel binding site could be pivotal for *RACK1* promoter regulation (Fig. 2D). Accordingly, MCF7 transfected with Δ7 and subsequently treated with 10 µM BAY 11-7085 resulted in a significant reduction of luciferase activity (Fig. 2E). Similarly, nandrolone and testosterone-BSA-FITC significantly decreased Δ7 luciferase activity. This supports the idea that the previously demonstrated impairment of c-Rel nuclear translocation (Fig. 2C) is related to RACK1 down-regulation, as also confirmed by protein expression (Fig. 2F).

Nandrolone and testosterone-BSA-FITC mAR-mediated effects on RACK1 expression were confirmed in MDA-MB-231 cells (Fig. 2G–I; Suppl. Fig. S3C), a metastatic and hormone-independent BC cell line lacking ARs, which is widely used as a TNBC model in vitro^49^. Once again, the PI3K/Akt/NF-κB pathway is involved in RACK1 expression as demonstrated by treatment with PI3K and IκBα inhibitors (Suppl. Fig. S3D, E).

Altogether, these results indicate that nandrolone exerts a strong negative regulation on RACK1 expression by antagonizing the PI3K/Akt/NF-κB pathway.

### Nandrolone decreased-RACK1 expression inhibits BC cell proliferation and migration

**Fig. 3:**
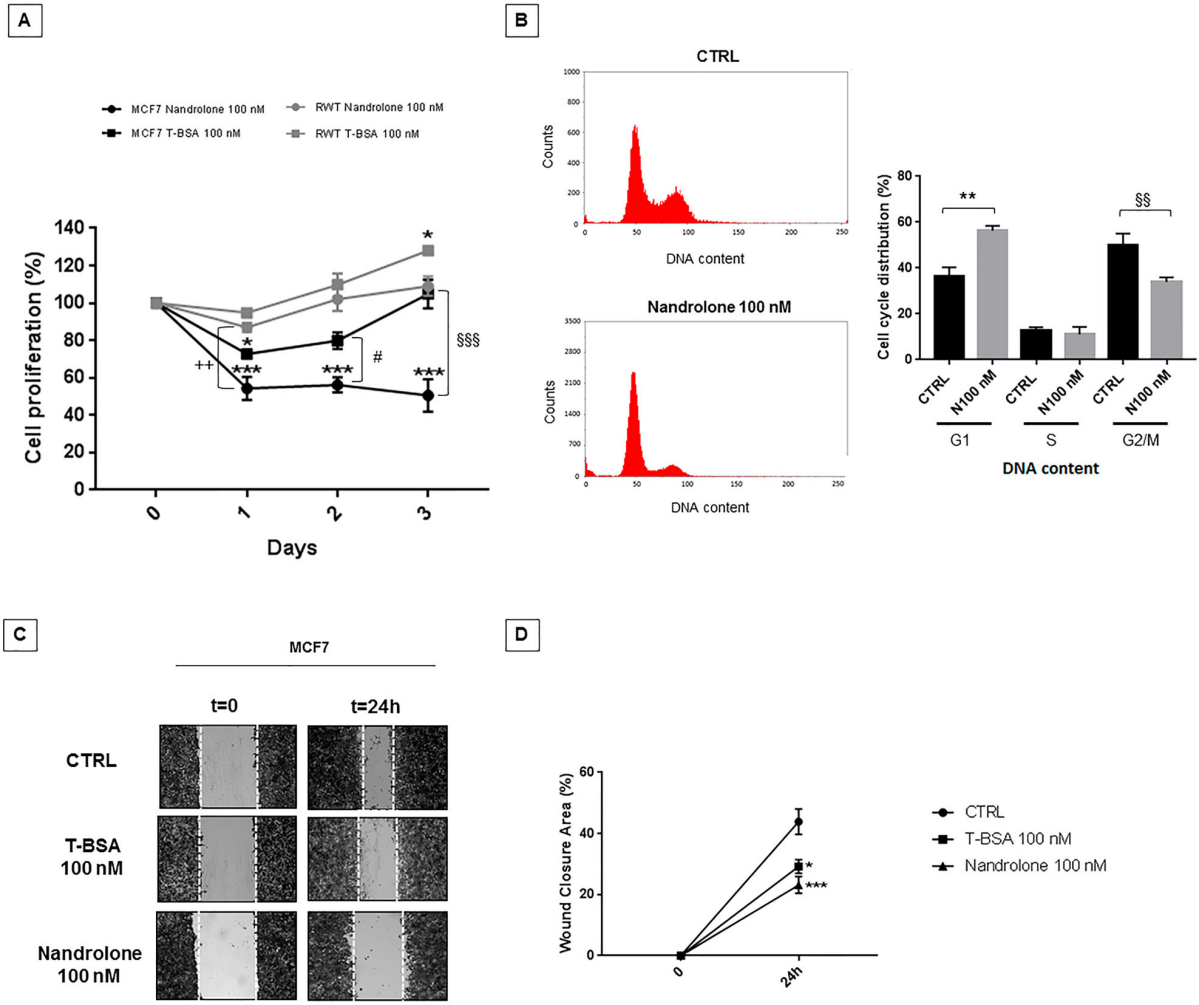
Nandrolone significantly reduces cell proliferation and migration. **A** MTT assay on MCF7 wild-type cells or over-expressing RACK1 (RWT) was performed to evaluate cell proliferation following 100 nM of testosterone-BSA-FITC (T-BSA) or nandrolone treatment for different timings (1, 2, and 3 days). Cells were treated at *t* = 0 and vehicle controls with DMSO < 0.1% (CTRL). Value bar in the graph represents the mean ± SEM of four independent experiments. The analysis was performed by two-way ANOVA with Tukey’s multiple comparisons test with *p < 0.05, **p < 0.01, ***p < 0.001 vs *t* = 0, ^#^*p* < 0.05 vs T-BSA at *t* = 2, ^§§§^*p* < 0.001 vs T-BSA at *t* = 3, ++*p* < 0.01 vs RWT at *t* = 1. **B** Effect of 100 nM Nandrolone treatment on the phases of the cell cycle. MCF7 cells were grown in complete medium and starved for 24 h. After 24 h of starvation, MCF7 cells were grown for 24 h in complete medium and then treated with DMSO < 0.1% (CTRL) or with 100 nM Nandrolone (N100 nM) for 24 h. The percentage of cells in each phase of the cell cycle was determined by flow cytometry after 24 h. The results are shown as the percentage of cells in each phase of the cell cycle. Data were expressed as mean ± SEM. from three independent experiments. The analysis was performed with Bonferroni’s multiple comparisons test, with **p < 0.01 vs CTRL G1, ^§§^*p* < 0.01 vs CTRL G2. **C**–**D** Effect of testosterone-BSA-FITC or nandrolone on MCF7 cells migration by scratch wound healing assay, performed as described in materials and methods. MCF7 cells were cultured with DMSO < 0.1% (CTRL) or with 100 nM of T-BSA or nandrolone and subsequently migration was analyzed at 24 h. **C** The image is a representative result. **D** Value bars in the graph represents the mean ± SEM of three independent experiments, in duplicate. The analysis was performed by two-way ANOVA with Tukey’s multiple comparisons test with *p < 0.05, ***p < 0.01 vs CTRL.

Testosterone-BSA-FITC or nandrolone treatments significantly inhibited MCF7 cell growth (Fig. 3A), in line with the RACK1 expression data (Fig. 2F) and the role played by the protein in cell proliferation (Suppl. Fig. S1A). Consistently, in MCF7 RWT cells, RACK1 over-expression rescued testosterone-BSA-FITC and particularly nandrolone treatments (Fig. 3A), confirming that cell growth inhibition is mediated by RACK1 downregulation. Nandrolone significantly increased the fraction of MCF7 cells in the G1 phase with a consequent decrease of cells in the G2/M phase (Fig. 3B), demonstrating that its anti-proliferative effect is associated with cell cycle alterations. Moreover, nandrolone or testosterone-BSA-FITC caused a significant decrease in BC cell migration, as shown by the reduction of wound area closure compared to control cells (Fig. 3C, D).

### OXER1 mediates the rapid antagonistic effect of androgens

**Fig. 4:**
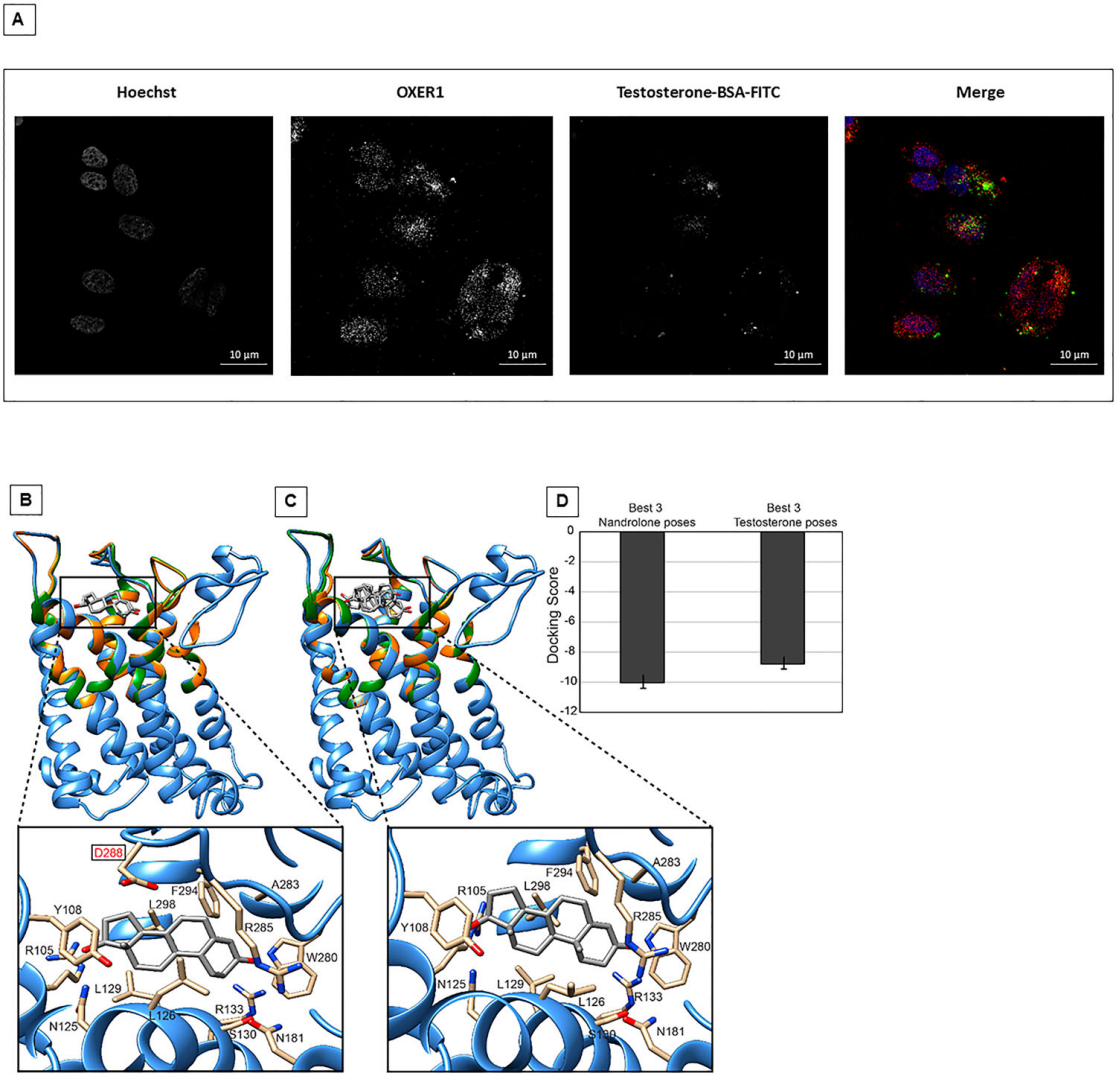
Structural model of OXER1 and molecular docking with nandrolone and testosterone. **A** Interaction between OXER1 and testosterone-BSA FITC in MCF7 cells. Acquisition by confocal microscope SP5 (for details see materials and methods). **B**–**C** Ribbon representation of the structural model of OXER1. The region of the receptor deputed to interact with the nandrolone or testosterone and considered flexible along the docking calculations is colored in orange and green respectively. For both molecules the 10 best docking poses are shown, while the insets report only the best one. **D** Mean value of the docking score for nandrolone and testosterone calculated on the best three poses.

Accumulating evidence indicates that androgens exert membrane-initiated actions leading to the modulation of cancer cell growth and metastasis. Three different G protein-coupled receptors (GPCRs) have been reported to mediate the membrane-initiated androgen effects: the G protein-coupled receptor family C group 6 member A (GPRC6A)^51–53^, the zinc transporter member 9 protein (ZIP9/SLC39A9)^54^, and the G protein-coupled OXER1^32,47^. Interestingly, testosterone acts agonistically on the first two receptors whereas it antagonizes OXER1-mediated actions^32,55^, which activate the PI3K/Akt and the focal adhesion kinase (FAK) signaling pathways^47^, promoting cell survival, adhesion and migration. Since OXER1 is expressed at high levels in MCF7 cells^32^ and our data demonstrate that nandrolone and testosterone-BSA FITC exert an antagonistic effect on survival and migration, we investigated OXER1 involvement in the two processes. As expected, testosterone-BSA FITC binds to OXER1 as demonstrated by in silico molecular docking data (Fig. 4) according to literature immunofluorescence data^44^.

### Nandrolone and testosterone binding mode on OXER1

The results so far discussed indicate a significant difference in the antagonist effects exerted by nandrolone and testosterone on OXER1. Indeed, despite the structural similarity of the two compounds, which differ only for a methyl group, nandrolone was endowed with a stronger activity. Thus, in order to provide a rationale for this difference in antagonist activity, we docked the two compounds in the binding pocket following modeling of the OXER1 three-dimensional (3D) structure (Fig. 4). The docking was performed using the induced fit protocol of the software Glide that confers flexibility to the lateral chains of the amino acids composing the binding pocket. In this way, we can obtain a more accurate prediction of the protein-ligand interaction mode.

Visual inspection of the ten docking poses obtained for each molecule (i.e., nandrolone e testosterone) clearly shows that nandrolone is always characterized by the same binding mode in all the best poses (Fig. 4B), while testosterone displays a larger variability in the binding mode (Fig. 4C). Furthermore, the absence of a methyl group on nandrolone C_10_ slightly decreases its steric hindrance compared to testosterone, allowing a deeper accessibility to the OXER1 binding site (Fig. 4). However, the network of interactions established with OXER1 is conserved between the two compounds, with a main difference regarding the interaction with residue D288, which is located on the extracellular loop three (ELIII) (Fig. 4B, C) and connects the two transmembrane (TM) helices 6 and 7. The ability of nandrolone to enter the cavity more deeply allows it to interact with ELIII, while this does not happen in the case of testosterone.

Finally, the docking score, which represents an indirect measure of the binding energy of the three best poses ranked for each compound, shows that nandrolone has a better mean score than testosterone, −10 ± 0.4 vs −8.5 ± 0.5 (Fig. 4D). These data further confirm the experimental evidence reported above.

### OXER1 role in cancer cell proliferation and migration through RACK1 expression regulation

**Fig. 5:**
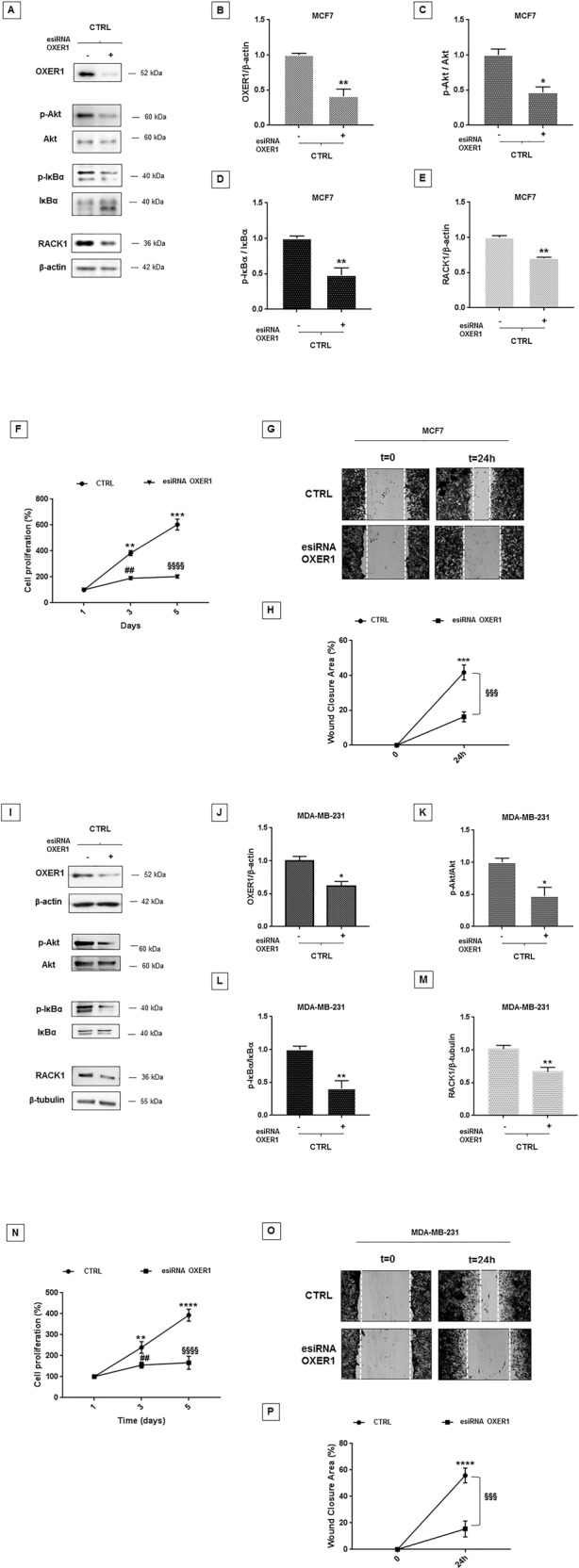
OXER1 silencing affects RACK1 expression with consequent inhibition of BC cell proliferation and migration. **A**–**E** MCF7 cells were silenced for 48 h with OXER1 siRNA to investigate OXER1-related PI3K/Akt pathway. **A** The image is a representative Western Blot. **B**–**E** Results are shown as OXER1/β-actin (**B**), p-Akt/Akt (**C**), p-IκBα/IκBα (**D**), and RACK1/β-actin (**E**) ratios ± SEM of four independent experiments. Statistical analysis was performed with Student’s *t*-test, with *p < 0.05, **p < 0.01. **F**–**H** Cellular proliferation and migration in MCF7 OXER1 silenced and unsilenced cells was performed as described in “Materials and methods”. **F** MTT assay on MCF7 silenced and unsilenced cells was performed to evaluate cell proliferation for different timings (1, 3 and 5 days). Value bar in the graph represents the mean ± SEM of three independent experiments. Statistical analysis was performed by two-way ANOVA with Tukey’s multiple comparisons test with **p < 0.01, ***p < 0.001 vs CTRL at *t* = 1, ^##^*p* < 0.01 vs CTRL at *t* = 3, ^§§§§^*p* < 0.0001 vs CTRL at *t* = 5. **G** The image is a representative result. **H** Value bar in the graph represents the mean ± SEM of three independent experiments, in duplicate of wound healing area in OXER1 silenced and unsilenced MCF7 cells. The analysis was performed by two-way ANOVA with Tukey’s multiple comparisons test with ***p < 0.001 vs CTRL at *t* = 0, ^§§§^*p* < 0.001 vs CTRL at *t* = 24 h. **I**–**M** MDA-MB-231 cells were silenced for 48 h with OXER1 siRNA to investigate OXER1-related PI3K/Akt pathway. **I** The image is a representative Western Blot. **J**–**M** Results are shown as OXER1/β-actin (**J**), p-Akt/Akt (**K**), p-IκBα/IκBα (**L**) and RACK1/β-tubulin (**M**) ratios ± SEM of three independent experiments. Statistical analysis was performed with Student’s *t*-test, with *p < 0.05, **p < 0.01. **N**–**P** Cellular proliferation and migration in MDA-MB-231 OXER1 silenced and unsilenced cells was performed as described in materials and methods. **N** MTT assay on MDA-MB-231 silenced and unsilenced cells was performed to evaluate cell proliferation for different timings (1, 3, and 5 days). Value bars in the graph represents the mean ± SEM of three independent experiments. Statistical analysis was performed with by two-way ANOVA with Tukey’s multiple comparisons test with **p < 0.01, ****p < 0.0001 vs CTRL at *t* = 1, ^##^*p* < 0.01 vs CTRL at *t* = 3, ^§§§§^*p* < 0.0001 vs CTRL at *t* = 5. **O** The image is a representative result. **P** Value bar in the graph represents the mean ± SEM of three independent experiments. The analysis was performed by two-way ANOVA with Tukey’s multiple comparisons test with ****p < 0.0001 vs CTRL at *t* = 0, ^§§§^*p* < 0.001 vs CTRL at *t* = 24 h.

OXER1 role in BC progression was confirmed by esiRNA, a mixture of siRNA oligos for the silencing approach, as OXER1 knockdown induced a significant down-regulation of the PI3K/Akt/NF-κB pathway and a substantial decrease in RACK1 expression (Fig. 5A–E). In addition, MCF7 cells silenced for OXER1 showed a significant decline of cell growth and migration (Fig. 5F–H), suggesting a functional link between RACK1 and OXER1.

Similarly, OXER1 silencing in MDA-MB-231, a BC cell line that significantly expresses OXER1^32^, causes a significant down-regulation of RACK1 expression due to impairment of the PI3K/Akt/NF-κB pathway (Fig. 5I–M). In accordance with our previous data^5^, decreased expression of RACK1 reduced cell proliferation and migration (Fig. 5N–P).

Altogether, these results demonstrate OXER1 involvement in MCF7 and MDA-MB-231 cells proliferation/migration and provide insights into the underlying signaling pathway.

### OXER1 and RACK1 mRNA expression in mammary tumors

**Fig. 6:**
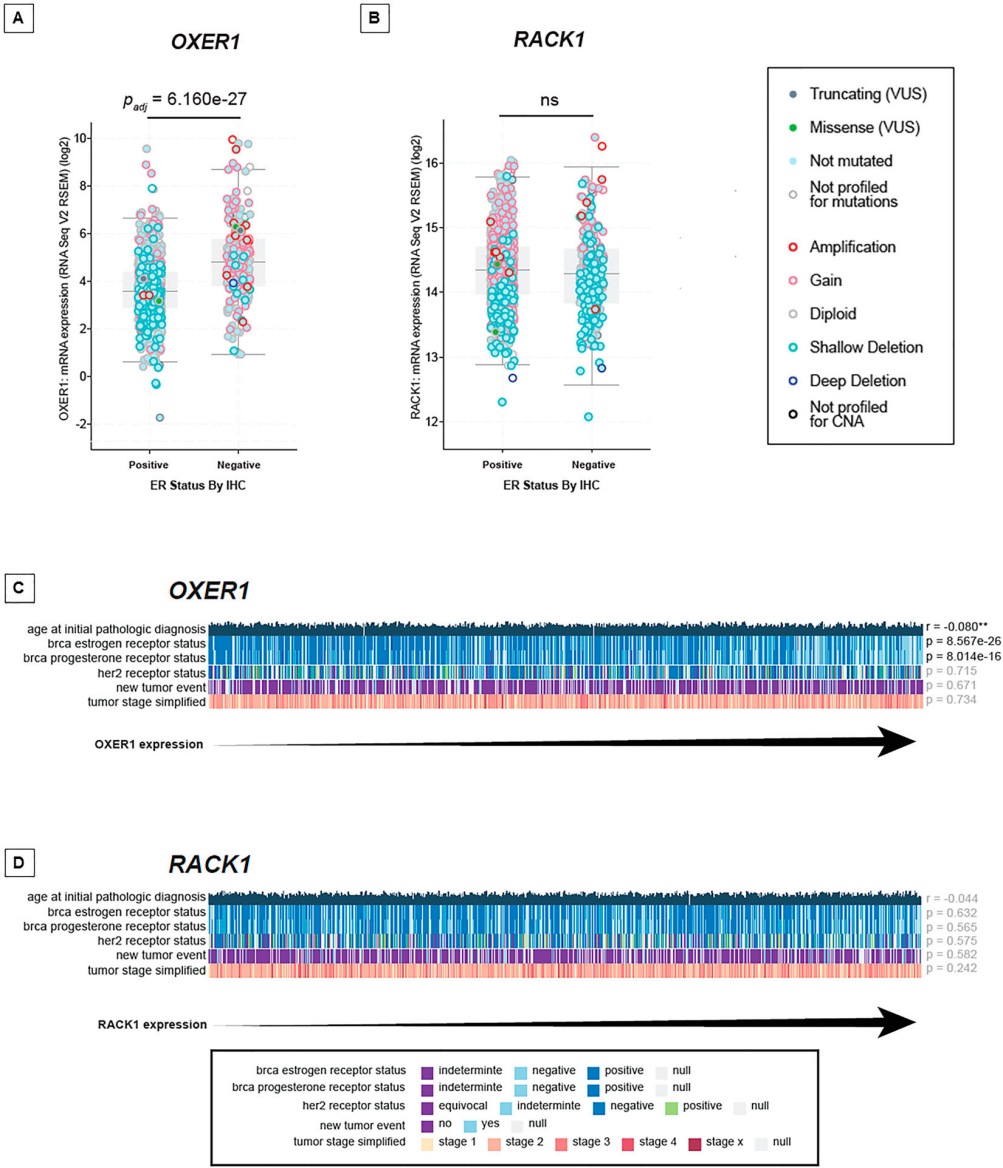
OXER1 mRNA expression in breast cancer subtypes. **A**–**B** Normalized expressions values for OXER1 (left) and RACK1 (right) were retrieved from TCGA (Firehose database) using cBioportal,and stratified according to Estrogen-receptor status. Provided is a legend with annotations respective to copy number and mutational status of the indicated genes. Statistical significance was assessed using Wilcoxon sum-rank test and adjusted for multiple comparison using Bonferroni correction. **C**–**D** Provided are clinical features (Age at diagnosis, Tumor stage, new tumor event, ER/PR/Her2 status) and their association to increasing expression levels of either OXER1 (top) or RACK1 (bottom) mRNAs. A detailed legend is provided at the bottom. Data were retrieved from TCGA using Mexpress portal (mexpress.be).

Our data support the idea that the OXER1/RACK1 pathway plays an oncogenic role in BC and they demonstrate that androgens downregulate the two proteins in BC cell lines. Thus, we evaluated the expression levels of OXER1 and RACK1 transcripts in estrogen-receptor-positive (ER^+^) and estrogen-receptor-negative (ER^−^) mammary tumors using the RNA-sequencing data available in the TCGA database. The data obtained were correlated with the copy number variation (CNV) and the mutation status of the two corresponding genes. The levels of OXER1 mRNA are significantly lower in ER^+^ compared to ER^-^ mammary tumors (Fig. 6A). This is likely to be due to the fact that the genome of ER^+^ tumors shows a high frequency of shallow deletions of the OXER1 gene. In contrast, the RACK1 mRNA expression levels do not show statistically significant differences in ER^+^ and ER^−^ cases (Fig. 6B). We performed further quantitative correlation studies between the expression levels of OXER1/RACK1 mRNAs and the following parameters: age of diagnosis, ER-positivity, PR-positivity, HER2-positivity, incidence of relapse (new tumor event) and tumor stage (Fig. 6C). As expected, OXER1 mRNA levels show a significant inverse correlation with ER-positivity and PR-positivity. More importantly, the transcript amounts are inversely correlated with the age of diagnosis. In other words, OXER1 mRNA is over-expressed in BC diagnosed at relatively young age, which is consistent with the idea that a large fraction of these cases are characterized by a triple-negative and ER^−^ phenotype. Once again, similar correlations are not observed in the case of RACK1 (Fig. 6D). Finally, the amounts of OXER1 and RACK1 mRNAs expressed in single tumors show a low but significant *r* correlation-value, indicating the existence of a weak co-regulation (Suppl. Fig. S4).

Given the potential role of OXER1 and RACK1 in BC progression, we evaluated the possibility that expression of the two corresponding mRNAs represent a useful predictive marker of survival for specific subtypes of mammary tumors. Hence, we determined possible correlations between OS and the levels of OXER1/RACK1 mRNAs in the ER^+^, HER2^+^ and triple-negative (TN) BC subgroups (Suppl. Fig S5). To conduct these analyses we divided the TCGA breast cancers in OXER1-high or RACK1-high and OXER1-low or RACK1-low tumors according to a threshold value represented by the median expression levels of the two transcripts (Suppl. Fig. S5). The results of this analysis demonstrate that there is no significant difference in the OS of OXER1-high and OXER1-low tumors as well as RACK1-high and RACK1-low tumors, regardless of the mammary tumor phenotype. The results are validated by comparison of the OS observed in tumors belonging to the first and last quartile in terms of both OXER1 and RACK1 mRNA expression. Thus, neither OXER1 nor RACK1 mRNAs seem to be useful predictive biomarkers for BC progression and lethality. However, a wider panel of patients needs to be analyzed to confirm this preliminary observation, especially in TNBC, and immunohistochemical analysis need also to be carried out. Indeed, a correlation with RACK1 expression, clinical state and histological grade was found in primary breast carcinomas^2–4^, providing evidence that RACK1 positive staining is related to a worse clinical outcome. RACK1 was also found to significantly discriminate between healthy controls and early stage BC patients^56–58^ thereby suggesting RACK1 as a possible superior predictor of BC prognosis and an independent prognosis related factor^2–4^. However, studies to evaluate RACK1 as a BC biomarker are still in their infancy and much validation work remains to be done^6^.

## Discussion

**Fig. 7:**
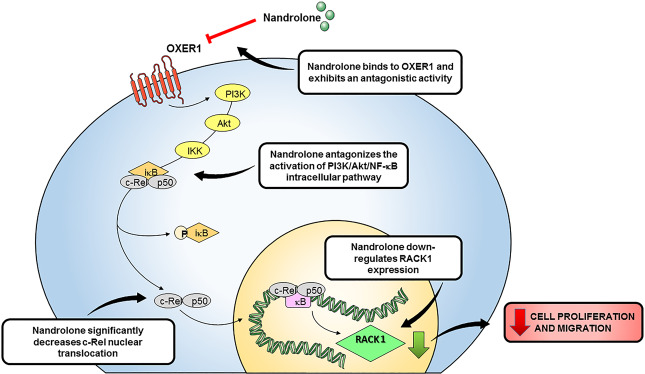
Schematic representation of nandrolone-antagonizing effects on OXER1-initiated pathwaw in BC cells. The binding of nandrolone to OXER1 antagonizes the PI3K/Akt/NF-κB pathway leading to a significant decrease of c-Rel nuclear translocation through a strong decline in RACK1expression, which inhibits cell proliferation and mi-initiated PI3K/Akt/ NF-κB pathway and RACK1 expression in BC cells (see text for more details).

BC is a hormone-dependent disease that encompasses biologically and clinically different tumor types^59^. The role of androgens in BC development and progression is still a matter of debate^1^, although androstanes and their structural analogs are endowed with significant anti-proliferative activity against different BC cells^23,60^. In this context, we demonstrated that nandrolone strongly impairs MCF7 and MDA-MB-231 cell proliferation and migration through an AR-independent mechanism since, in presence of the well-known AR inhibitor flutamide^42^, it is still able to downregulate RACK1 expression. This is in accordance with emerging evidence that mAR belonging to GPCR family are reported and proposed to mediate membrane-initiated androgen effects^32,51–55^. Our data provide the first evidence that nandrolone effect is due to an AR-independent mechanism, which involves downregulation of RACK1, a protein controlling BC progression and positively regulated by the PI3K/Akt/NF-κB pathway^3–5^. We provided evidence that testosterone-BSA-FITC and nandrolone antagonize the PI3K/Akt/NF-κB pathway leading to a significant decrease of c-Rel nuclear translocation through a strong decline in RACK1 expression, which inhibits cell proliferation and migration (Fig. 7). Although NF-κB plays an important role in normal mammary gland morphogenesis, its increased levels are commonly observed in BCs as a consequence of the constitutive expression of the NF-κB subunits (e.g., c-Rel, p65 and p50)^61–63^. Indeed, stimulation of the NF-κB signaling pathway promotes angiogenic neovascularization, epithelial-to-mesenchymal transition (EMT) and increases cancer cell stemness, leading to chemoresistance, radioresistance and endocrine resistance. By converse, NF-κB inhibition increases the sensitivity of cancer cells to chemo- and radio-therapy and consequently disease-free survival in BC patients^64^.

AR-independent nandrolone activity was confirmed in the MDA-MB-231 cell line, where androgens have been reported to trigger membrane-initiated steroid signaling (MISS) and gene transcription^50^. Many of the genes regulated by testosterone-BSA control the activity of DNA binding inhibitors, exert dominant negative effects on the helix-loop-helix proteins pathways and related transcription factors, preventing cancer cell-growth/-survival and angiogenesis^50,65^. In addition, testosterone-BSA decreases the levels of cytoskeletal proteins, cytokines, inflammation-related molecules and their correlated-pathways, including several interleukins (IL2, IL4, IL6, and IL12) and TNF-related molecules. All this suggests modulation of a variety of cytokines, chemokines and growth factors, which are produced in the local tumor environment and promote tumor progression. In addition, testosterone-BSA treatment induces pro-apoptotic and anti-proliferative effects in both hormone-responsive or resistant BC cells^50^. Finally, literature indicates that testosterone-BSA and testosterone modify the expression of largely different sets of genes, in line with the idea that the two compounds act via an AR-independent and an AR-dependent mechanism, respectively^50^. This is exactly what we observe in MCF7 cells where the anti-tumor action of testosterone-BSA is OXER1 and RACK1 dependent.

Many of the non-classical and cell surface dependent actions exerted by androgens are mediated by novel mARs, i.e., GPCRC6A, ZIP9/SLC39A9 and OXER1. Testosterone acts as an agonist of ZIP9/SLC39A9 and GPRC6A, although the latter protein is a non-specific membrane AR^32,53^. On the contrary, testosterone is an OXER1 antagonist. As OXER1 inhibits some of the major signaling pathways (FAK, PI3K/Akt, p38α) controlling cell growth, is likely to represent a new mediator of the anti-proliferative and anti-migratory actions exerted by androgens in cancer cells^47,50^. Indeed, our data demonstrate that antagonistic effects on the OXER1 receptor mediate RACK1 downregulation induced by testosterone-BSA and nandrolone. This results in a significant reduction of BC cell proliferation and migration, which is known to be promoted by the interaction between FAK and RACK1^4,6,38,66^. Hence, we provide evidence that OXER1 is a determinant of BC cell proliferation and migration in both luminal BC subtype and TNBC models *via* activation of the PI3K/Akt/NF-κB pathway. In particular, our findings provide new insights on RACK1 transcriptional regulation in our TNBC cell model. In accordance with our literature data reporting in RACK1 promoter region the presence of a GRE (glucocorticoid response element) site close to the c-Rel site here investigated^5,11^ and the possible synergism between GR and NF-κB in BC^67^, we hypothesize that in MDA-MB-231 cells NF-κB and GR could work synergistically for RACK1 transcriptional regulation, considering that we previously demonstrated GR involvement in RACK1 regulation in this same TNBC model^5^.

Interestingly, NF-κB/GR cross-talk promotes BC development/progression and it has been suggested as a potential target for BC therapy^67^.

The data provided in the present study indicate that OXER1 represents a novel link between androgens and their AR-independent action. In addition, they suggest that androgenic molecules lacking a classic AR-dependent profile and characterized by effects mediated by membrane ARs (e.g., nandrolone) can be exploited to reduce BC cell proliferation and migration. In fact, our molecular docking data reveal that nandrolone is a strong OXER1 antagonist, which suggests that OXER1 is a promising molecular target of androgen derivatives. With respect to this, it is worthwhile mentioning that the literature data indicate significant OXER1 expression differences between non-cancerous and cancerous tissues^32^. Interestingly, our study shows a significant inverse correlation between OXER1 mRNA levels and ER-positivity, PR-positivity or age of diagnosis in BC. These last observations are consistent with the idea that a large fraction of the cases defined by high OXER1 expression is characterized by a triple-negative and ER^−^ phenotype. However, in prostate cancer (PC), OXER1 transcripts are lower in tumor than in normal tissue, pointing out that epigenetic elements may also need to be considered in the expression of OXER1 in PC^47^.

In conclusion, our data support the idea that androgen derivatives tailored to antagonize OXER1 activation pathway may represent a promising and rational agents for the personalized treatment of TNBC.

## Supplementary information

Supplemental Material

## References

[1] Song JL, Chen C, Yuan PJ, Sheng-Rong Sun SR (2016). Progress in the clinical detection of heterogeneity in breast cancer. Cancer Med..

[2] Cao XX (2010). A superior independent predictor for poor clinical outcome in breast cancer. Int. J. Cancer.

[3] Cao XX (2010). RACK1 promotes breast carcinoma proliferation and invasion/metastasis in vitro and in vivo. Breast Cancer Res. Treat..

[4] Cao XX (2011). RACK1 promotes breast carcinoma migration/metastasis via activation of the RhoA/Rho kinase pathway. Breast Cancer Res. Treat..

[5] Buoso E (2019). Cortisol-induced SRSF3 expression promotes GR splicing, RACK1 expression and breast cancer cells migration. Pharm. Res..

[6] Buoso E. et al. Ribosomes as nexus between translation and cancer progression: focus on ribosomal RACK1 in Breast Cancer. *Br. J. Pharmacol*. 10.1111/bph.15218 (2020). 10.1111/bph.15218 32726469

[7] Li JJ, Xie D (2015). RACK1, a versatile hub in cancer. Oncogene.

[8] Duff D, Aideen Long A (2017). Roles for RACK1 in cancer cell migration and invasion. Cell Signal.

[9] Buoso E (2017). Transcriptional regulation of RACK1 and modulation of its expression: role of steroid hormones and significance in health and aging. Cell Signal.

[10] R2: Genomics Analysis and Visualization Platform (http://r2.amc.nl).

[11] Del Vecchio I (2009). Functional mapping of the promoter region of the GNB2L1 human gene coding for RACK1 scaffold protein. Gene.

[12] Racchi M (2017). Role of hormones in the regulation of RACK1 expression as a signaling checkpoint in immunosenescence. Int. J. Mol. Sci..

[13] Buoso E (2011). Opposing effects of cortisol and dehydroepiandrosterone on the expression of the receptor for activated C kinase 1: implications in immunosenescence. Exp. Gerontol..

[14] Buoso E (2017). Role of spliceosome proteins in the regulation of glucocorticoid receptor isoforms by cortisol and dehydroepiandrosterone. Pharm. Res..

[15] Buoso E (2017). The scaffold protein RACK1 is a target of endocrine disrupting chemicals (EDCs) with important implication in immunity. Toxicol. Appl. Pharm..

[16] Buoso E (2020). Effect of estrogen-active compounds on the expression of RACK1 and immunological implications. Arch. Toxicol..

[17] Torre LA (2017). Global cancer in women: burden and trends. Cancer Epidemiol. Biomark. Prev..

[18] Giovannelli P (2018). The androgen receptor in breast cancer. Front Endocrinol..

[19] López-Marure R, Contreras PG, Dillon JS (2011). Effects of dehydroepiandrosterone on proliferation, migration, and death of breast cancer cells. Eur. J. Pharm..

[20] López-Marure R (2016). Dehydroepiandrosterone inhibits events related with the metastatic process in breast tumor cell lines. Cancer Biol. Ther..

[21] Niro S, Hennebert O, Morfin R (2010). New insights into the protective effects of DHEA. Horm. Mol. Biol. Clin. Investig..

[22] Schneider G (2016). Stereocontrolled synthesis of the four 16-hydroxymethyl-19-nortestosterone isomers and their antiproliferative activities. Steroids.

[23] Gyovai A (2018). Antiproliferative properties of newly synthesized 19-nortestosterone analogs without substantial androgenic activity. Front. Pharm..

[24] Iványi Z (2012). Synthesis of D-ring-substituted (5’R)- and (5’S)-17β-pyrazolinylandrostene epimers and comparison of their potential anticancer activities. Steroids.

[25] Ajduković JJ (2013). 17(E)-picolinylidene androstane derivatives as potential inhibitors of prostate cancer cell growth: antiproliferative activity and molecular docking studies. Bioorg. Med. Chem..

[26] Ajduković JJ (2015). Synthesis, structural analysis and antitumor activity of novel 17α-picolyl and 17(E)-picolinylidene A-modified androstane derivatives. Bioorg. Med. Chem..

[27] Acharya PC, Bansal R (2014). Synthesis and antiproliferative activity of some androstene oximes and their O-alkylated derivatives. Arch. Pharm..

[28] Cui J (2015). Synthesis, characterization and antitumor activities of some steroidal derivatives with side chain of 17-hydrazone aromatic heterocycle. Steroids.

[29] Jakimov DS (2015). Androstane derivatives induce apoptotic death in MDA-MB-231 breast cancer cells. Bioorg. Med. Chem..

[30] Powell WS, Joshua Rokach J (2015). Biosynthesis, biological effects, and receptors of hydroxyeicosatetraenoic acids (HETEs) and oxoeicosatetraenoic acids (oxo-ETEs) derived from arachidonic acid. Biochim. Biophys. Acta.

[31] Stepniewski TM (2018). Synthesis, molecular modelling studies and biological evaluation of new oxoeicosanoid receptor 1 agonists. Bioorg. Med. Chem..

[32] Kalyvianaki K (2019). Membrane androgen receptors (OXER1, GPRC6A AND ZIP9) in prostate and breast cancer: a comparative study of their expression. Steroids.

[33] Romano N, Veronese M, Manfrini N, Zolla L, Ceci M (2019). Ribosomal RACK1 promotes proliferation of neuroblastoma cells independently of global translation upregulation. Cell Signal.

[34] Buoso E (2013). Modulation of Rack-1/PKCβII signalling by soluble AβPPα in SH-SY5Y cells. Curr. Alzheimer Res..

[35] Buoso E (2012). AβPP intracellular C-terminal domain function is related to its degradation processes. J. Alzheimers Dis..

[36] Tang F (2012). MicroRNA-125b induces metastasis by targeting STARD13 in MCF-7 and MDA-MB-231 breast cancer cells. PLoS ONE.

[37] Grolla AA (2015). Nicotinamide phosphoribosyltransferase (NAMPT/PBEF/visfatin) is a tumoural cytokine released from melanoma. Pigment Cell Melanoma Res..

[38] Serrels B (2010). A complex between FAK, RACK1, and PDE4D5 controls spreading initiation and cancer cell polarity. Curr. Biol..

[39] Lee, A. V., Oesterreich, S. & Davidson, N. E. MCF-7 cells–changing the course of breast cancer research and care for 45 years. *J. Natl. Cancer Inst*. **107**, djv073 (2015). 10.1093/jnci/djv073 25828948

[40] Kiely M (2017). RACK1 stabilises the activity of PP2A to regulate the transformed phenotype in mammary epithelial cells. Cell Signal.

[41] Sasano H, Miki Y, Nagasaki S, Suzuki T (2009). In situ estrogen production and its regulation in human breast carcinoma: from endocrinology to intracrinology. Pathol. Int..

[42] De Abrew KN (2016). Grouping 34 chemicals based on mode of action using connectivity mapping. Toxicol. Sci..

[43] Hatzoglou A (2005). Membrane androgen receptor activation induces apoptotic regression of human prostate cancer cells in vitro and in vivo. J. Clin. Endocrinol. Metab..

[44] Kampa M (2005). Opposing effects of estradiol- and testosterone-membrane binding sites on T47D breast cancer cell apoptosis. Exp. Cell Res..

[45] Kampa M (2006). Activation of membrane androgen receptors potentiates the antiproliferative effects of paclitaxel on human prostate cancer cells. Mol. Cancer Ther..

[46] Kampa M, Castanas E (2006). Membrane steroid receptor signaling in normal and neoplastic cells. Mol. Cell Endocrinol..

[47] Kalyvianaki K (2017). Antagonizing effects of membrane-acting androgens on the eicosanoid receptor OXER1 in prostate cancer. Sci. Rep..

[48] Akter R, Hossain MZ, Kleve MG, Gealt MA (2012). Wortmannin induces MCF-7 breast cancer cell death via the apoptotic pathway, involving chromatin condensation, generation of reactive oxygen species, and membrane blebbing. Breast Cancer.

[49] Jang HY (2020). 15d-PGJ2 inhibits NF-κB and AP-1-mediated MMP-9 expression and invasion of breast cancer cell by means of a heme oxygenase-1-dependent mechanism. BMB Rep..

[50] Notas G, Pelekanou V, Castanas E, Kampa M (2010). Conjugated and non-conjugated androgens differentially modulate specific early gene transcription in breast cancer in a cell-specific manner. Steroids.

[51] Pi M (2008). GPRC6A null mice exhibit osteopenia, feminization and metabolic syndrome. PLoS ONE.

[52] Pi M, Parrill AL, Quarles LD (2010). GPRC6A mediates the non-genomic effects of steroids. J. Biol. Chem..

[53] Pi M, Quarles LD (2012). GPRC6A regulates prostate cancer progression. Prostate.

[54] Thomas P, Pang Y, Dong J, Berg AH (2014). Identification and characterization of membrane androgen receptors in the ZIP9 zinc transporter subfamily: II. Role of human ZIP9 in testosterone-induced prostate and breast cancer cell apoptosis. Endocrinology.

[55] Thomas P (2019). Membrane androgen receptors unrelated to nuclear steroid receptors. Endocrinology.

[56] Lacombe J, Mangé A, Bougnoux AC, Prassas I, Solassol J (2014). A multiparametric serum marker panel as a complementary test to mammography for the diagnosis of node-negative early-stage breast cancer and DCIS in young women. Cancer Epidemiol. Biomark. Prev..

[57] Lacombe J (2013). Identification and validation of new autoantibodies for the diagnosis of DCIS and node negative early-stage breast cancers. Int. J. Cancer.

[58] Eswaran J (2012). Transcriptomic landscape of breast cancers through mRNA sequencing. Sci. Rep..

[59] Secreto G, Girombelli A, Krogh V (2019). Androgen excess in breast cancer development: implications for prevention and treatment. Endocr. Relat. Cancer.

[60] Kampa M, Notas G, Castanas E (2017). Natural extranuclear androgen receptor ligands as endocrine disruptors of cancer cell growth. Mol. Cell Endocrinol..

[61] Cogswell PC, Guttridge DC, Funkhouser WK, Baldwin AS (2000). Selective activation of NF-kappaB subunits in human breast cancer: potential roles for NF-kappa B2/p52 and for Bcl-3. Oncogene.

[62] Nakshatri H, Bhat-Nakshatri P, Martin DA, Goulet RJ, Sledge GW (1997). Constitutive activation of NF-kappaB during progression of breast cancer to hormone-independent growth. Mol. Cell Biol..

[63] Sovak MA (1997). Aberrant nuclear factor-kappaB/Rel expression and the pathogenesis of breast cancer. J. Clin. Invest..

[64] Wang W, Nag SA, Zhang R (2015). Targeting the NFκB signaling pathways for breast cancer prevention and therapy. Curr. Med. Chem..

[65] Ling MT, Wang X, Zhang X, Wong YC (2006). The multiple roles of Id-1 in cancer progression. Differentiation.

[66] Deevi RK, Cox OT, O’Connor R (2014). Essential function for PDLIM2 in cell polarization in three-dimensional cultures by feedback regulation of the β1-integrin-RhoA signaling axis. Neoplasia.

[67] West DC (2018). Discovery of a glucocorticoid receptor (GR) activity signature using selective GR antagonism in ER-negative breast cancer. Clin. Cancer Res..

