# Supplementary material for: OXER1 and RACK1-associated pathway: a promising drug target for breast cancer progression
Source: Oncogenesis. 2020 Dec 11;9(12):105. doi: 10.1038/s41389-020-00291-x (PMC7732991; doi:10.1038/s41389-020-00291-x)
Supplement: Supplementary file 1 — Supplemental Material [file 41389_2020_291_MOESM1_ESM.pdf]

SUPPLEMENTARY INFORMATION

This file contains all the supplementary figures, tables and the relative legends mentioned in the manuscript.

Supplementary figures and figure legends

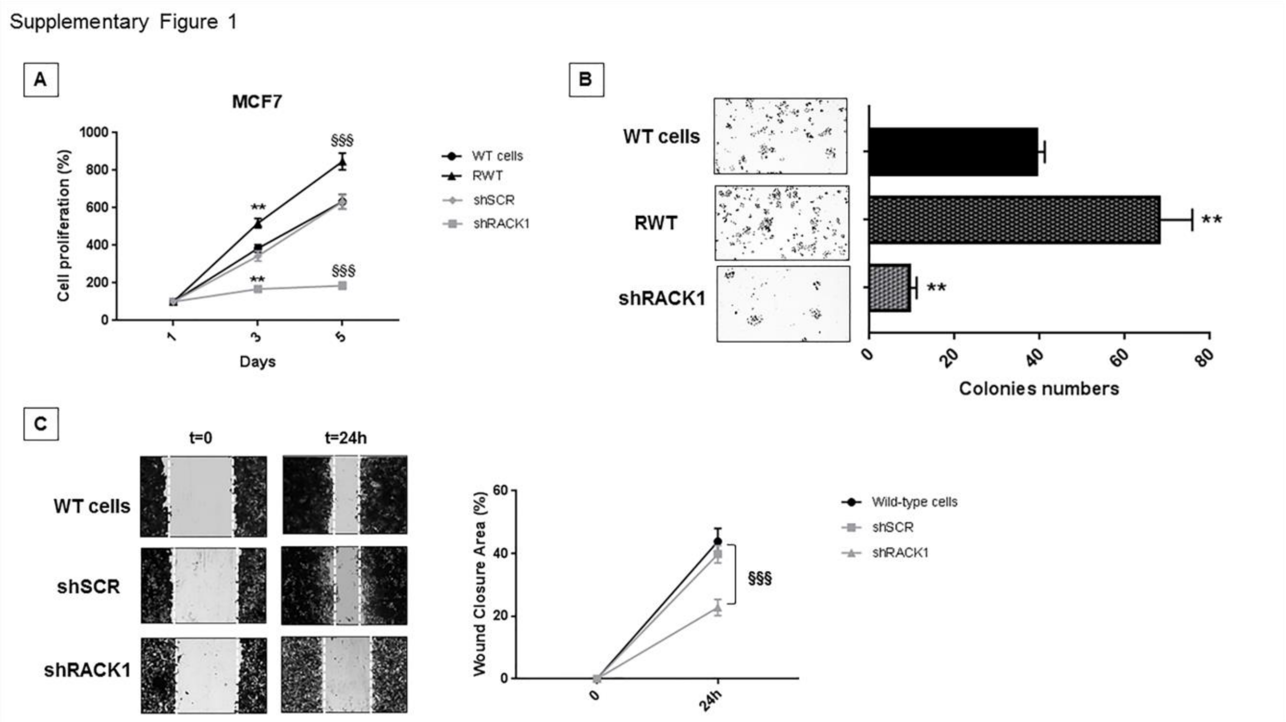

Suppl. Figure 1. MCF7 cell proliferation and migration is correalted with RACK1 expression **A.** To evaluate RACK1 expression on MCF7 cell proliferation, MTT assay was performed on wild-type cells (WT cells) or stably transfected with sh scramble (shSCR), silenced for RACK1 (shRACK1) or over-expressing RACK1 (RWT). Value bar in the graph represents the mean ± SEM of three independent experiments. The analysis was performed by two-way ANOVA with Tukey's multiple comparisons test with $^{**}p < 0.01$ vs WT cells at t=3, §§§ $p < 0.001$ vs WT cells at t=5. **B.** MCF7 WT, shRACK1 or RWT cells were seeded in MW6 plates and colonies were colored and counted after 120 h according to literature data [1]. Value bar in the graph represents the mean ± SEM of three independent experiments, in duplicate. Statistical analysis was performed with Dunnett's multiple comparison test, with ** $p < 0.01$. **C.** Evaluation of cell migration in MCF7 WT, shSCR or shRACK1 by scratch wound healing assay, performed as described in materials and methods. Value bar in the graph represents the mean ± SEM of three independent experiments, in duplicate. Wound healing area were analyzed by two-way ANOVA with Bonferroni's multiple comparisons test with §§§ $p< 0.001$ vs WT cells at t=24h.

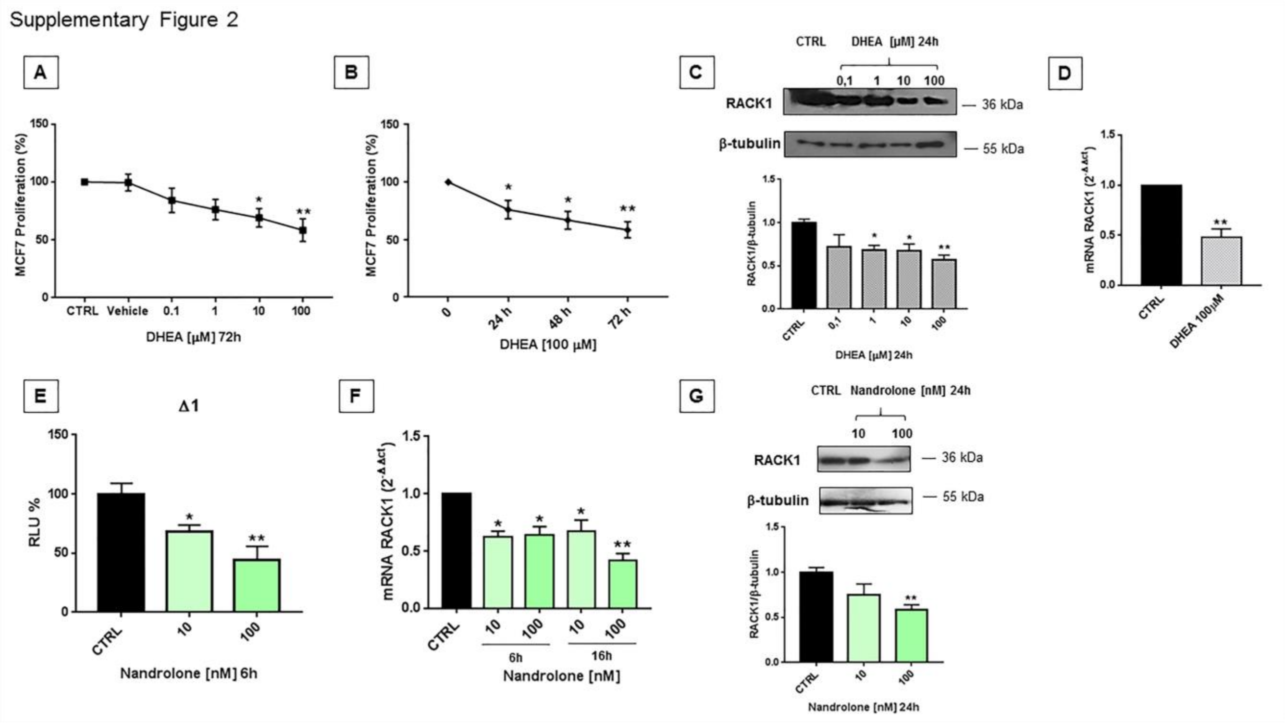

Suppl. Figure 2. MCF7 cell proliferation and androgens activity on RACK1 expression

A-B. MTT assay was performed in MCF7 to evaluate cell proliferation after DHEA treatment. **A.** DHEA effect at different concentration (0.1, 1, 10 and 100 μM) after 72 h of treatment. **B**. 100 μM DHEA at different timings (0, 24, 48 and 72 h). In **A-B**, each value in the graph represents the mean ± SEM of four independent experiments. Statistical analysis was performed with Dunnett's multiple comparisons test with $*p < 0.05$ and $**p < 0.01$. **C**. MCF7 cells were treated with 0.1, 1, 10 and 100 μM DHEA for 24 h. Vehicle controls were treated with DMSO < 0.1% (CTRL). The image is a representative Western Blot. Results are shown as RACK1/β-tubulin ratio ± SEM of four independent experiments. Statistical analysis was performed with Dunnett's multiple comparison test, with $*p < 0.05$ and $**p < 0.01$. **D.** MCF7 cells were treated with DMSO < 0.1% (CTRL) or 100 μM DHEA for 16h and RACK1 mRNA expression analysis was performed by real-time PCR as described in materials and methods. Significance was set at $p < 0.05$ by the Student's *t*-test ($**p < 0.01$). **E-G.** Effect of nandrolone on RACK1 expression. **E**. Δ1 represented the entire 2-kb region 5′ of the human *RACK1* gene as discussed in the text**.** MCF7 cells were transfected with Δ1 and subsequently treated with 10 and 100 nM nandrolone for 6 h. Vehicle controls were treated with DMSO < 0.1% (CTRL). For each reporter construct, luciferase activity was expressed as RLU% and compared to CTRL values assumed at 100%. Each bar represents the mean ± SEM of three independent experiments, in triplicate. Statistical analysis was performed with Dunnett's multiple

comparison test, with *p < 0.05 and **p < 0.01. **F.** MCF7 cells were treated with DMSO < 0.1% (CTRL), 10 and 100 nM nandrolone for 6 h and 16h and RACK1 mRNA expression analysis was performed by real-time PCR as described in materials and methods. Each bar represents the mean ± SEM of four independent experiments. Statistical analysis was performed with Dunnett's multiple comparison test, with *p < 0.05 and **p < 0.01. **G.** MCF7 cells were treated with DMSO < 0.1% (CTRL). 100 nM Nandrolone for 24 h. Cells were collected, lysed and RACK1 protein levels were analyzed through Western Blot. The image is a representative Western Blot. Results are shown as RACK1/β-tubulin ratio ± SEM. Statistical analysis was performed with Dunnett's multiple comparison test, with **p < 0.01.

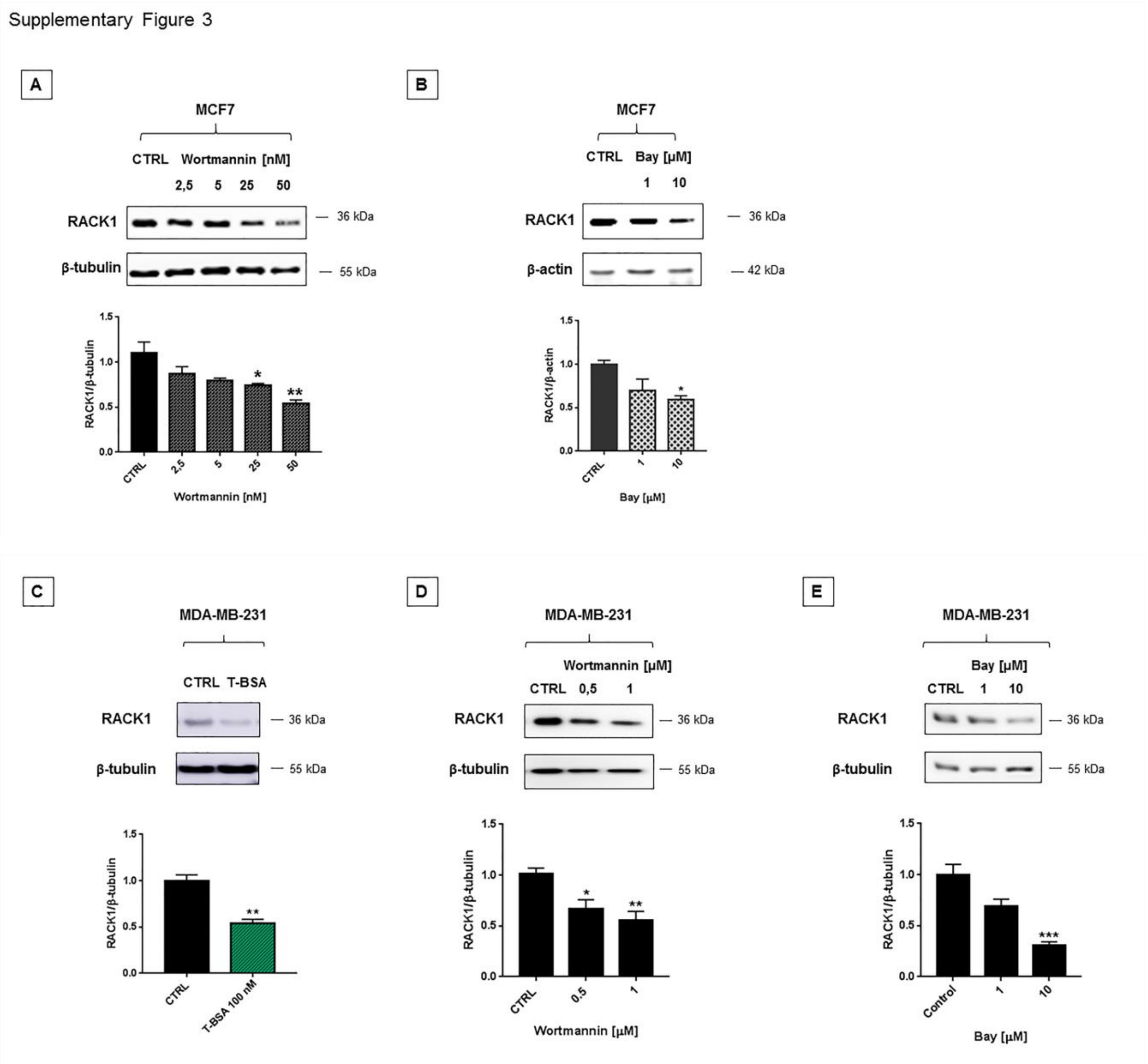

Suppl. Figure 3. RACK1 expression is regulated by PI3K/Akt/NF-κB pathway

A-B. MCF7 cells were treated with increasing concentration of wortmannin (2,5 -50 nM) (A) or BAY 11-7085 (1 - 10 µM) (B). Vehicle controls were treated with DMSO < 0.1% (CTRL). Cells were collected, lysed and RACK1 protein levels were analyzed through Western Blot. The images are representative Western Blots. Results are shown as RACK1/β-tubulin ratio ± SEM of three (A) or four (B) independent experiments. Statistical analysis was performed with Dunnett's multiple comparison test, with *p < 0.05, **p < 0.01. **C.** MDA-MB-231 cells were treated for 24 h with 100 nM Testosterone-BSA-FITC (T-BSA). Vehicle controls were treated with DMSO < 0.1% (CTRL). The image is a representative Western Blots. Results are shown as and RACK1/β-tubulin ratios ± SEM of three independent experiments. Significance was set at p < 0.05 by the Student's *t*-test (** p < 0.01). **D-E.** MDA-MB-231 cells were treated with increasing concentration of wortmannin (0,5 and 1µM) (D) or BAY 11-7085 (1 - 10 µM) [2] (E). Vehicle controls were treated with DMSO < 0.1% (CTRL). Cells were collected, lysed and RACK1 protein levels were analyzed through Western Blot. The images are representative Western Blots. Results are shown as RACK1/β-tubulin ratio ± SEM of three (D) or four (E) independent experiments.

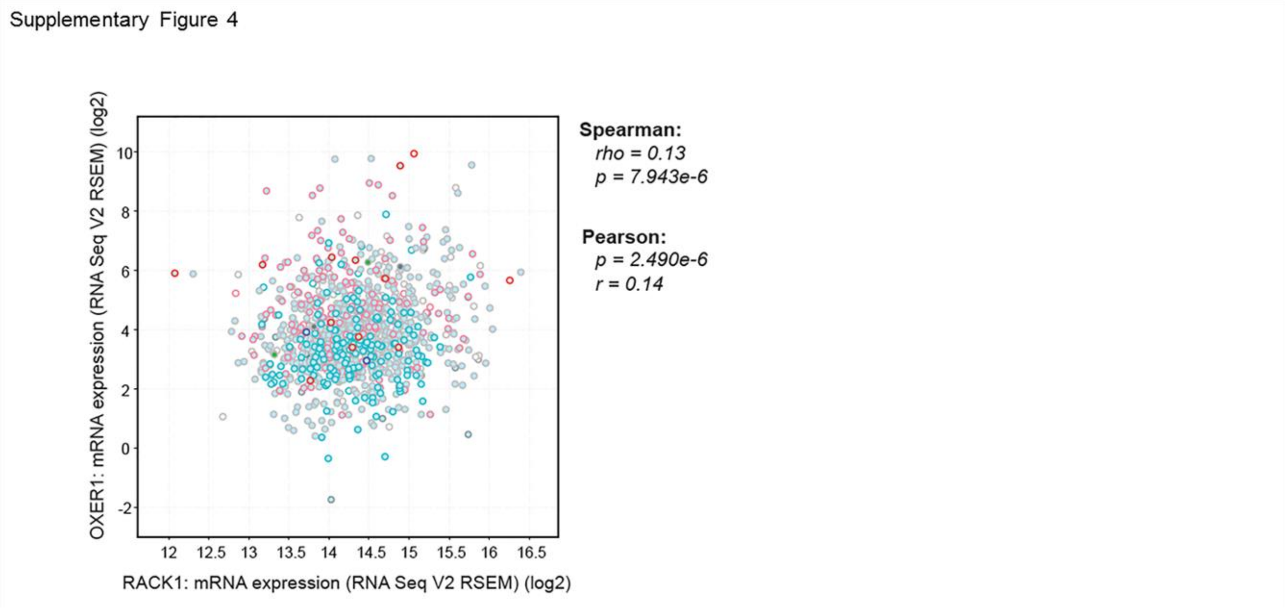

Suppl. Figure 4. Correlation between OXER1 and RACK1 mRNA expression

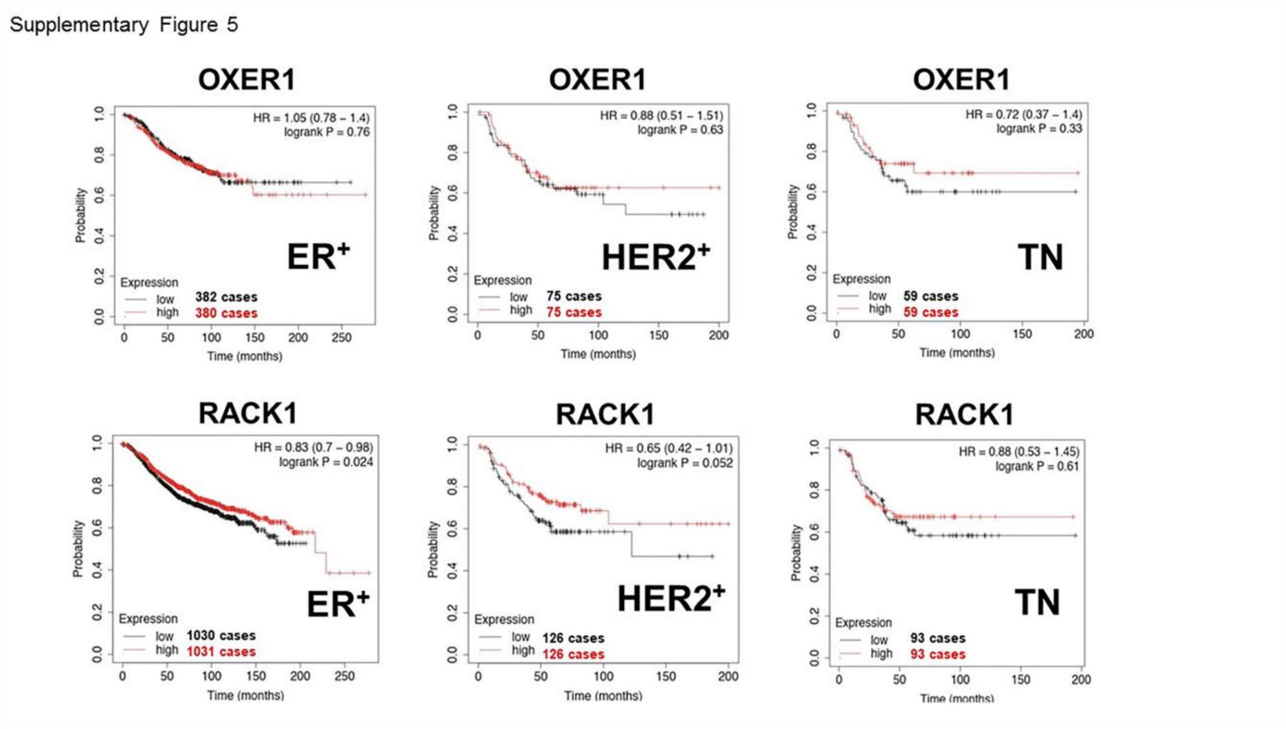

Suppl. Figure 5. Survival analysis

Survival information from an integrated cohort of 3955 breast cancer patients were obtained using Kaplan-Meier Plotter (kmplot.com). Patients with highest expression levels (1st quartile) were compared to those showing lowest levels of expression (4th Quartile) of either OXER1 (top) or RACK1 (bottom) mRNAs. Comparisons were performed separately for ER+ tumors (left), Her2+ tumors (middle) and TN tumors only (right). Provided are Hazard Ratios (HR) and P-values as deterimined by LogRank test.

Supplementary Tables

Suppl. Table 1. Sequence similarity between OXER1 and the chosen templates

Helix	Template suggestion(s)	Sequence similarity
TMH1	hOPRK1 -> 4DJH	63.33
TMH2	hHRH1 -> 3RZE	57.14
TMH3	zLpar6 -> 5XSZ	70.59
TMH4	hFFAR1 -> 4PHU	33.33
TMH5	sRho -> 2Z73	46.67
TMH6	hP2Y12 -> 4NTJ	48.39
TMH7	hF2RL1 -> 5NDD	57.69
H8	hF2RL1 -> 5NDD	45.45

References - Supplementary Materials

1. Romano N, Veronese M, Manfrini N, Zolla L, Ceci M. Ribosomal RACK1 promotes proliferation of neuroblastoma cells independently of global translation upregulation. Cell Signal. 2019;53:102-110. doi:10.1016/j.cellsig.2018.09.020.

2. Smith SM, Lyu YL, Cai L. NF-κB affects proliferation and invasiveness of breast cancer cells by regulating CD44 expression. PLoS One. 2014;9(9):e106966. Published 2014 Sep 3. doi:10.1371/journal.pone.0106966
